# Comprehensive Exploration of Tumor Microenvironment Modulation Based on the ESTIMATE Algorithm in Bladder Urothelial Carcinoma Microenvironment

**DOI:** 10.3389/fonc.2022.724261

**Published:** 2022-02-14

**Authors:** Ji Chen, Boyu Lv, Yating Zhan, Kai Zhu, Rongrong Zhang, Bo Chen, Yan Jin, Yeping Li, Jianjian Zheng, Changyong Lin

**Affiliations:** ^1^ Key Laboratory of Diagnosis and Treatment of Severe Hepato-Pancreatic Diseases of Zhejiang Province, The First Affiliated Hospital of Wenzhou Medical University, Wenzhou, China; ^2^ Department of Urology, The First Affiliated Hospital of Wenzhou Medical University, Wenzhou, China; ^3^ Department of General Surgery, Wenzhou Hospital of Traditional Chinese Medicine Affiliated to Zhejiang Chinese Medical University, Wenzhou, China

**Keywords:** bladder urothelial carcinoma, immune infiltrates, immune/stromal scores, tumor microenvironment, prognostic signature

## Abstract

Recently, the tumor microenvironment (TME) has been reported to be closely related to the tumor initiation, progression, and prognosis. Bladder urothelial carcinoma (BLCA), one of the most common subtypes of bladder cancer worldwide, has been associated with increased morbidity and mortality in the past decade. However, whether the TME status of BLCA contributes to the prediction of BLCA prognosis still remains uncertain. In this study, the ESTIMATE algorithms were used to estimate the division of immune and stromal components in 406 BLCA samples downloaded from The Cancer Genome Atlas database (TCGA). Based on the comparison between ESTIMATE scores, the differentially expressed genes (DEGs) were selected. Using the univariate Cox regression analysis, prognosis-related DEGs were further identified (*p* < 0.05). The LASSO regression analysis was then used to screen 11 genes that were highly related to the TME of BLCA to generate a novel prognostic gene signature. The following survival analyses showed that this signature could effectively predict the prognosis of BLCA. The clinical value of this signature was further verified in an external cohort obtained from the First Affiliated Hospital of Wenzhou Medical University (*n* = 120). Based on the stage-correlation analysis and differential expression analysis, IGF1 and MMP9 were identified as the hub genes in the signature. Additionally, using CIBERSORT algorithms, we found that both IGF1 and MMP9 were significantly associated with immune infiltration. Collectively, a novel TME-related prognostic signature contributes to accurately predict the prognosis of BLCA.

## Introduction

Bladder urothelial carcinoma (BLCA), one of the most common subtypes of bladder cancer (BC) worldwide, has been associated with increased morbidity and mortality in the past decade (1, 2). Also, BLCA is the seventh most common cause of cancer-related mortality in the world. BLCA could be categorized into two distinctive types: muscle-invasive bladder cancer (MIBC) and nonmuscle-invasive bladder cancer (NMIBC), of which 25% represents MIBC and 75% represents NMIBC (3). Patients with NMIBC have a 50%–70% rate of relapse, with a 5-year survival rate >90%. However, after progressing to MIBC, the 5-year survival rate will drop to <50% (4). With the development of treatments and surgical techniques, patients with MIBC still have a poor prognosis with a 5-year survival rate of 5% (5, 6). Therefore, there is an urgent need to explore novel, effective biomarkers for the treatment of BLCA.

Recent studies have shown that the status of the tumor microenvironment (TME) is involved in the development of cancers (6). TME mainly contains tumor stromal cells and extracellular matrix (7). Increasing evidence has demonstrated that TME participates in the regulation of the invasiveness of tumor cells *via* chemotherapy, immune scape, etc. (8, 9). The types of nontumor components in TME mainly consist of immune cell and stromal cells, which play a key role in the diagnosis and prognosis of various human cancers (10). Immune cells have been reported to be associated with TME chemotaxis (11, 12). Moreover, stromal cells have been shown to be involved in tumor angiogenesis as well as extracellular matrix remodeling (13, 14). Notably, immune infiltration plays a crucial role in the modulation of TME status (15, 16).

Currently, to explore the novel TME-related prognostic signature for prognostic treatment of BLCA, there is an increase in interest in the evaluation of the stromal and immune components in the TME and tumor immune infiltration, especially in the comprehensive exploration of TME modulation. In this study, the stromal and immune scores were calculated *via* the ESTIMATE algorithm. The correlation analyses between ESTIMATE scores and clinical features revealed that TME status may be associated with the prognosis of BLCA. Based on the least absolute shrinkage and selection operator (LASSO) regression analysis and multivariate Cox regression analysis, a novel TME-related signature was generated to improve the prognosis prediction of BLCA. In addition, IGF1 and MMP9 were identified as the hub genes in the signature and used for the further immune infiltration analyses.

## Materials and Methods

### Data Collections

The cases of BLCA transcriptome data (FPKM normalized), including adjacent nontumorous samples and BLCA samples, were downloaded from The Cancer Genome Atlas (TCGA) (https://portal.gdc.cancer.gov). The relevant clinicopathological characteristics of BLCA patients were also downloaded from TCGA database (**Table 1**). According to each patient’s ID number, we matched the transcriptomic data and clinical features, and the data of the mismatched patients were removed. Finally, the gene expression profiles of 406 BLCA patients were obtained. An external cohort (named FAHWMU, *n* = 120) was obtained from the First Affiliated Hospital of Wenzhou Medical University (Wenzhou, China) to validate the clinical value of the prognostic signature. The collection of this cohort was reviewed and approved by the human research ethics committee of the First Affiliated Hospital of Wenzhou Medical University. The patients/participants provided their written informed consent to participate in this study.

**Table 1:** Clinical characteristics of patients in TCGA database.

Variables	TCGA (N = 406)
**Status**	
Alive	227
Dead	179
**Age (median)**	67
**Gender**	
Female	106
Male	300
**TNM stage**	
Stage I	2
Stage II	129
Stage III	140
Stage IV	133
Unknown	2
**Grade**	
Low	21
High	382
Unknown	3

### ESTIMATE Algorithms and Differential Expression Analyses

On the basis of the ESTIMATE algorithm, the relevant scores of infiltrating stromal cells and immune cells in BLCA patients were estimated (17). According to the median values of the immune and stromal scores, we divided the BLCA cases into two groups, the high group and the low group, respectively. We then obtained the heatmaps of the immune and the stromal scores using the R package named “heatmap.” The differential expression analyses were performed through the Wilcoxon test. The differentially expressed genes (DEGs) with *p* < 0.05 and | log fold change | >1.5 were filtered in the immune and stromal score groups. These genes with log fold change >1.5 were defined as upregulated genes, while genes with log fold change <−1.5 were defined as downregulated genes.

### Enrichment Analyses

The DEGs were used for enrichment analyses from Kyoto Encyclopedia of Genes and Genomes (KEGG) and Gene Ontology (GO) databases using “ggplot2,” “org.Hs.eg.db,” “enrichplot,” and “clusterProfiler” packages. The DEGs with both *p-* and *Q*-values <0.05 were considered the significantly enriched genes.

### Univariate Cox Regression Analysis and the Protein–Protein Interaction Network Construction

DEGs with *p* < 0.05 were screened as prognosis-related genes through the univariate Cox regression analysis. Using the Retrieval of Interacting Gene (STRING) database (https://www.string-db.org/), the protein–protein interaction (PPI) network was then obtained. The interactive relationships that were greater than 0.15 were used as the nodes. To better identify the hub genes, the Cytoscape software (version 3.8.2) (https://cytoscape.org/) was used to reconstruct the PPI network. The Gene Set Enrichment Analysis (GSEA) software (version 4.1.0) (http://www.gsea-msigdb.org/gsea/) was used to analyze all tumor cases through C7.all.v7.4.symbols.gmt gene sets.

### Generation of the Prognostic Signature

The LASSO regression analysis was performed among 45 DEGs regarding overall survival (OS) to remove the ones that were overfitted to the signature. The multivariate Cox regression analysis was then used to identify 11 hub genes related to the BLCA microenvironment, and their coefficients were obtained to calculate the risk score. The risk score was calculated according to the following computational formula: risk score = Ʃ (*β_i_* * Exp*_i_*) (*i* = 11). In addition, according to the median values of risk scores, we categorized the BLCA patients into the high-risk and the low-risk groups. The “survival” and “survival ROC” packages were used to generate the receiver operating characteristic curve (ROC) to predict the prognostic value of the signature. The log rank test was used to perform the Kaplan–Meier survival analysis between the high-risk and low-risk groups.

### Identification of the Hub Genes

The “ggpubr” and “limma” R packages were used to analyze the correlations between TNM stage and the genes of the signature. Then, the hub DEGs between adjacent nontumorous samples and BLCA samples were screened through differential expression analysis. Lastly, to determine the hub genes of the prognostic signature, the “Venndiagram” R package was used to identify the common genes between stage-correlation analyses and differential analyses.

### Immune Infiltration Analyses

In BLCA patients, the contents of immune-infiltrating cells were obtained *via* the CIBERSORT algorithm. Only samples with a CIBERSORT *p* < 0.05 were included for the next analysis. Subsequently, “ggExtra,” “vioplot,” “ggpubr,” and “gglot2” R packages were applied to perform the stage-correlation analyses. In the analysis of Pearson coefficient tests and Wilcoxon rank-sum, *p* < 0.05 was set as the criterion for a significant difference. In addition, immune correlation analysis was performed using the “limma” R package and Person’s correlation coefficients.

## Results

### The Correlation Between Clinicopathological Characteristics and the TME of BLCA Was Significant

**Figure 1:**
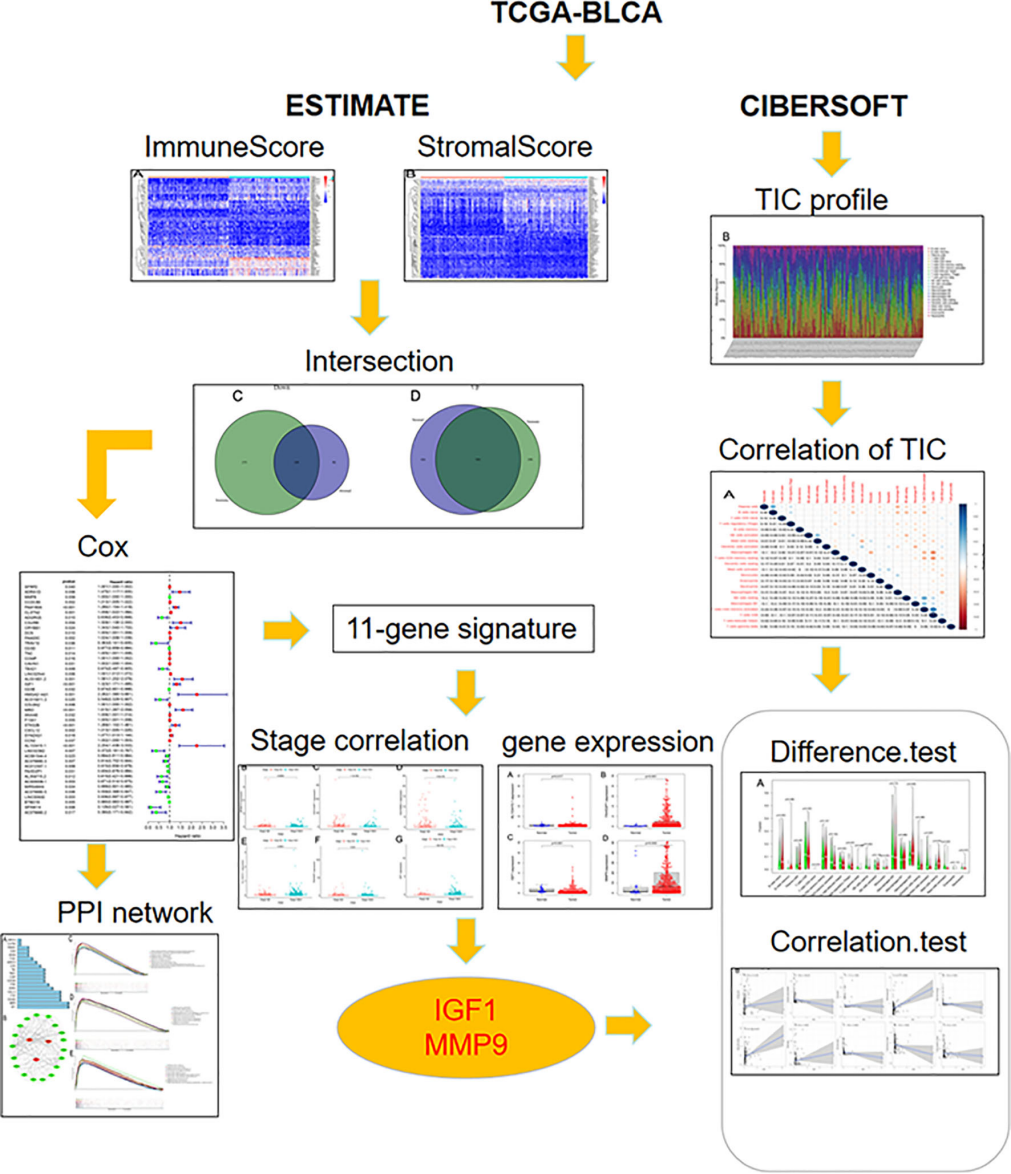
The overall workflow of this study.

**Figure 2:**
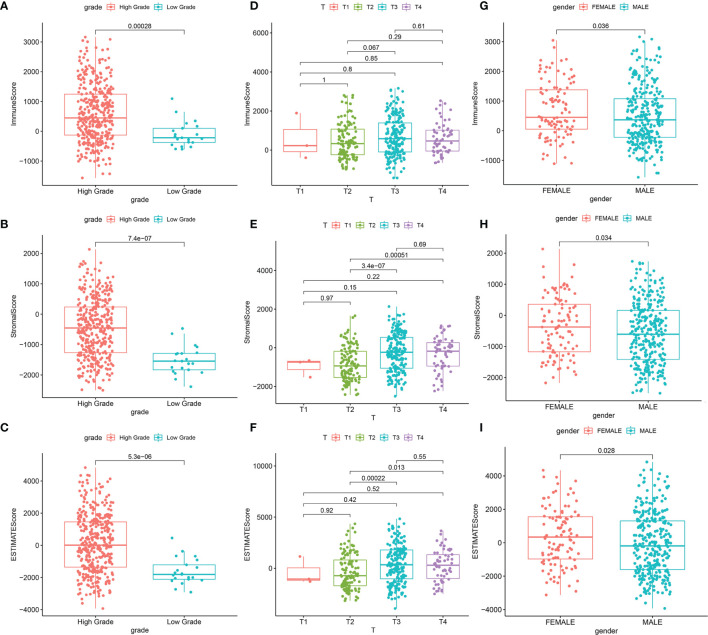
Significant correlation between clinicopathological characteristics and scores. **(A–C)** Correlation between the scores and grade. **(A)** Distribution of ImmuneScore in grade (*p* = 0.00028). **(B)** Distribution of StromalScore in grade (*p* = 7.4e−07). **(C)** Distribution of ESTIMATEScore in grade (*p* = 5.3e−06). **(D–F)** Correlation between scores and T classification. **(D)** Distribution of ImmuneScore in T classification. **(E)** Distribution of StromalScore in T classification. **(F)** Distribution of ESTIMATEScore in T classification. **(G–I)** Correlation between scores and gender. **(G)** Distribution of ImmuneScore in gender (*p* = 0.036). **(H)** Distribution of StromalScore in gender (*p* = 0.034). **(I)** Distribution of ESTIMATEScore in gender (*p* = 0.028).

The overall workflow of this study is shown in **Figure 1**. Recently, several studies have shown that a higher immune or stromal score indicates more immune or stromal components in the TME (18). Using the ESTIMATE algorithm, it was found that the ESTIMATE, the stromal, and the immune scores were significantly associated with gender and grade, especially for the T2 and T3 histologically graded disease (**Figures 2A**–**I**). The *p*-values of gender were 0.036, 0.034, and 0.028, respectively, while the *p*-values of grade were 0.00028, 7.4e−07, and 5.3e−06, respectively. In addition, the *p*-values between T2 and T3 were 0.067, 3.4e−07, and 0.00022, respectively. The stromal scores were also positively associated with N0 and N2 of histological grades (*p* = 0.0018). However, no significant relationships between the scores and other clinicopathological features were found (**Supplementary Figure S1**). These correlation analyses suggest that the stromal and immune scores may be associated with the progression of BLCA.

### A Total of 1,097 Genes Were Identified as DEGs

**Figure 3:**
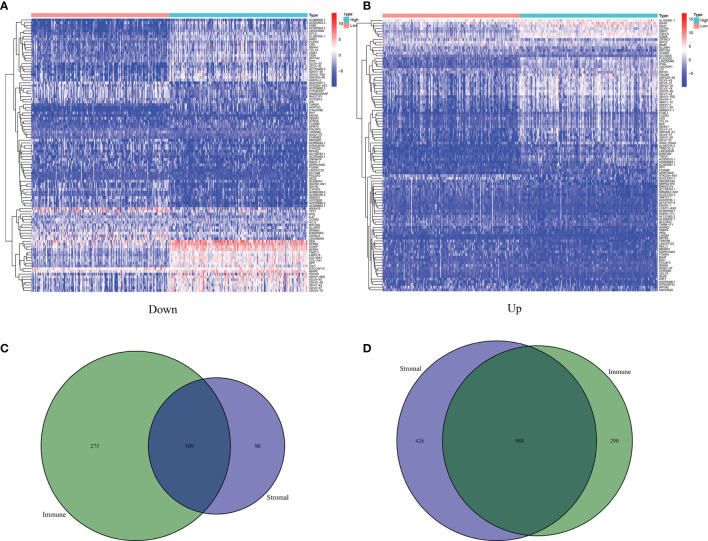
Identification of 1,027 DEGs between ImmuneScore and StromalScore. **(A)** Heatmap for DEGs in ImmuneScore, only showing the top 60 significantly DEGs (*p* < 0.05, fold change > 1.5). **(B)** Heatmap for DEGs in StromalScore, only showing the top 60 significant DEGs (*p* < 0.05, fold change > 1.5). **(C)** Venn plots showing 109 DEGs that were commonly upregulated both in ImmuneScore and StromalScore. **(D)** Venn plots showing 988 DEGs that were commonly downregulated both in ImmuneScore and StromalScore.

Based on the median values of the immune score as well as stromal score, all BLCA patients were divided into two groups. As shown in the heatmaps, DEGs were identified by the differential analyses (**Figures 3A, B**). On the basis of the median values of immune score, there were 1,662 DEGs including 384 downregulated genes and 1,278 upregulated genes. Similarly, based on the stromal score, 1,613 DEGs including downregulated 199 and upregulated 1,414 were identified. The common DEGs between the immune score group and the stromal score group were shown in the Venn diagram (**Figures 3C, D**). Finally, a total of 1,097 common DEGs were identified and used for the next studies.

### Immune-Related Pathways Were Enriched in the GO and KEGG Enrichment Analyses

**Figure 4:**
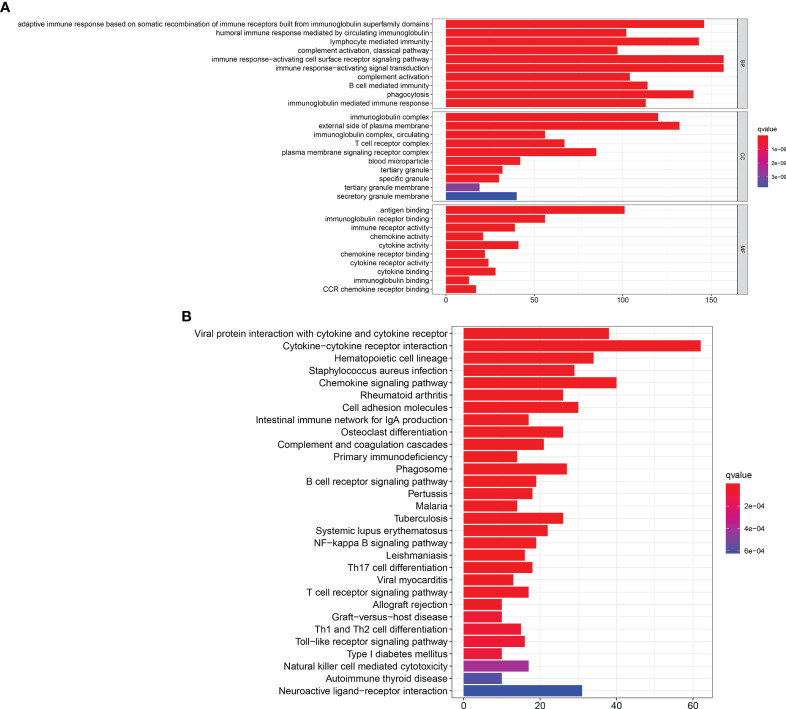
GO and KEGG enrichment analyses. **(A)** GO enrichment analysis for 1,097 DEGs, only both *p* and *q* < 0.05 were considered significant. **(B)** KEGG pathways enrichment analysis for 1,097 DEGs, only both *p* and *q* < 0.05 were considered significant.

The GO and KEGG pathway enrichment analyses were performed to select the potential molecular pathways among 1,097 DEGs. GO analysis showed that the DEGs were mainly enriched in humoral immune response mediated by circulating immunoglobulins, lymphocyte-mediated immunity, and immune response-activating cell surface receptor signaling pathway. These pathways were strongly associated with immune system (**Figure 4A**). Moreover, results of KEGG analysis indicated that the DEGs were significantly associated with cytokine–cytokine receptor interactions and immune pathways (B-cell receptor signaling pathway, T-cell receptor signaling pathway, and intestinal immune network for IgA production) (**Figure 4B**). Our results suggest that immune-related pathways may participate in the progression of BLCA.

### Identification of the Hub Genes

**Figure 5:**
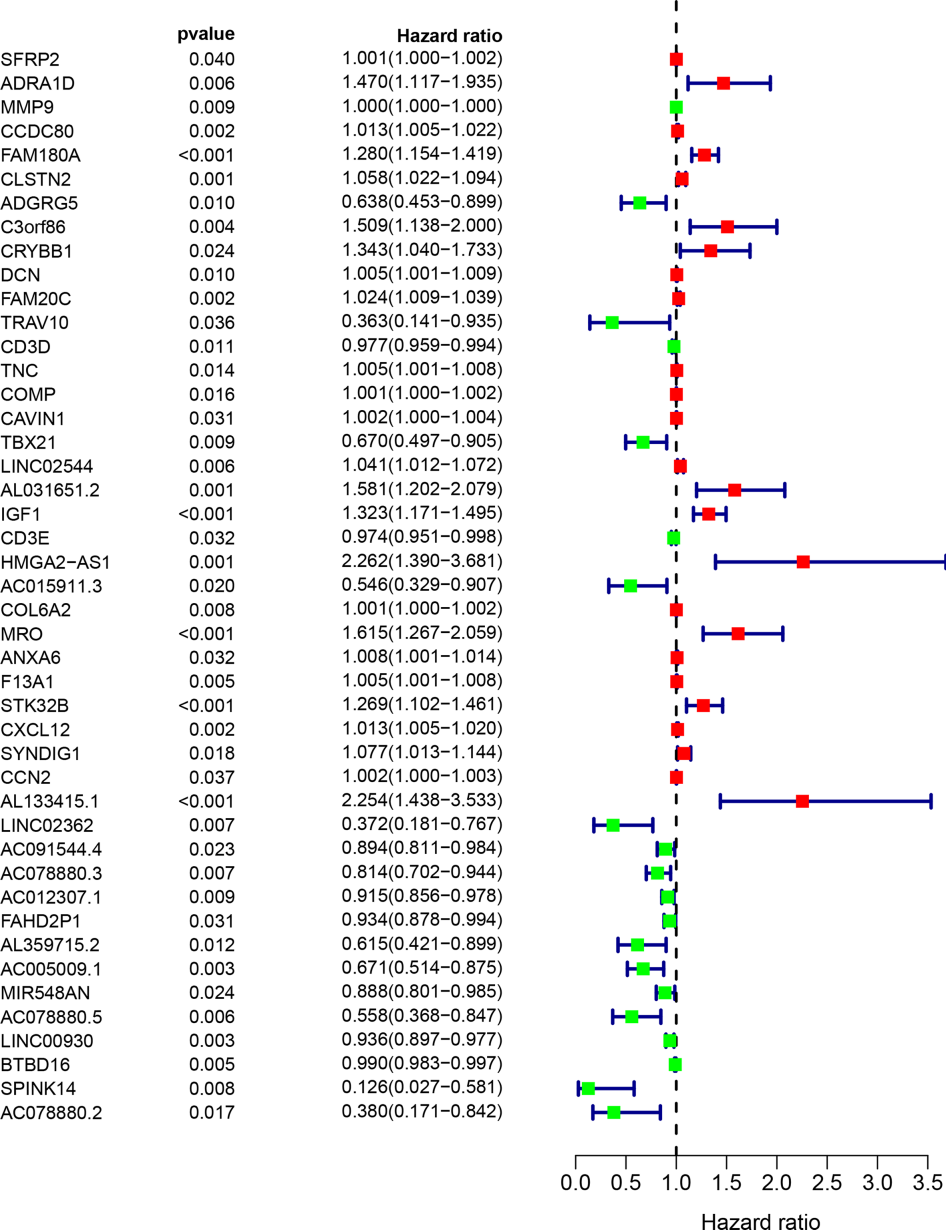
The univariate COX regression analysis of the DEGs. Only 45 DEGs were significantly correlated with prognosis (*p* < 0.05).

**Figure 6:**
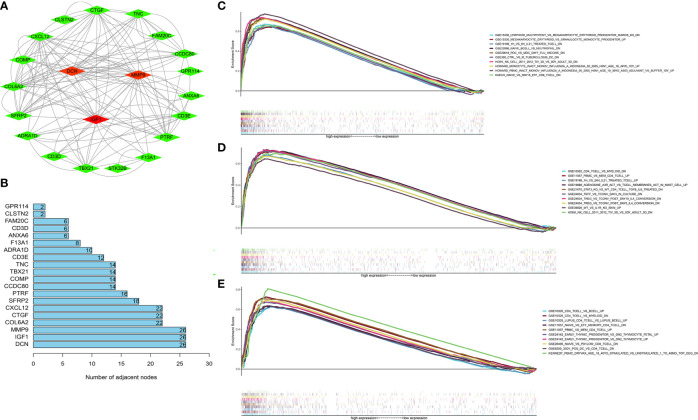
GSEA enrichment analyses of the hub genes. **(A)** The PPI network of 45 PRDEGs (coefficients > 0.15). **(B)** DCN, MMP9, and IGF1 had the most nodes in the PPI network. **(C–E)** The GSEA enrichment analyses of IGF1 **(C)**, MMP9 **(D)**, and IGF1 **(E)**.

Next, the 1,097 DEGs were exploited into univariate Cox regression analysis. Thus, 45 prognostic-related DEGs were identified (*p* < 0.05) (**Figure 5**). Using the SRING tool and Cytoscape software, the PPI network was constructed to explore their underlying interplays (**Figure 6A**). During PPI network, many genes with too few nodes (nodes < 2) were deleted. As shown in **Figure 6B**, DCN, IGF1, and MMP9 were identified as relatively remarkable nodes. Furthermore, GSEA enrichment analysis revealed that 3 immune-related pathways including the CD4 T-cell pathway, MEM CD4 T-cell pathway, and native B-cell pathway, may influence the progression of BLCA (**Figures 6C–E**).

### Construction and Verification of the Prognostic Signature

**Figure 7:**
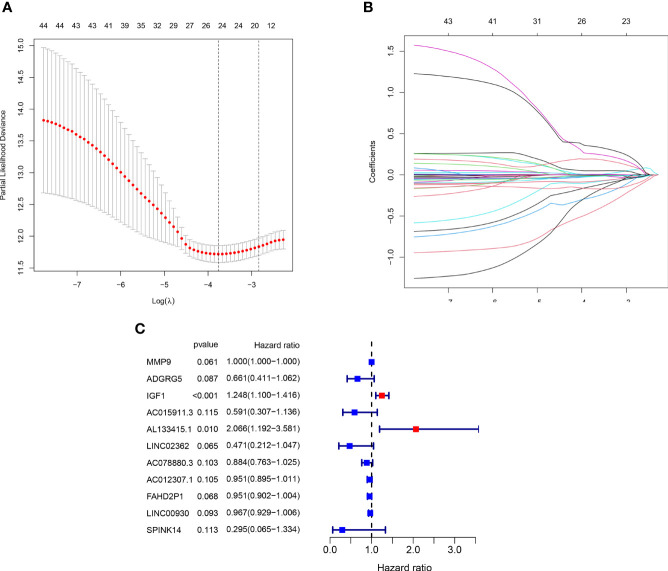
Generation of the prognostic risk signature. **(A, B)** The LASSO regression analysis of the 45 PRDEGs. **(C)** The Forest plot showing eight hub genes selected by the multivariate regression analysis.

The LASSO analysis was performed to avoid overfit of the signature (**Figures 7A, B**). Subsequently, 11 TME-related genes were identified by the multivariate Cox analysis (**Figure 7C**). The risk formula was calculated as:

Risk score = −0.414 * ADGRG5 + 0.001 * MMP9 + 0.222 * IGF1 − 0.526 * AC015911.3 + 0.726 * AL133415.1 − 0.753 * LINC02362 − 0.123 * AC078880.3 − 0.050 * AC012307.1 − 0.050 * FAHD2P1 − 0.034 * LINC00930 − 1.220 * SPINK14.

**Figure 8:**
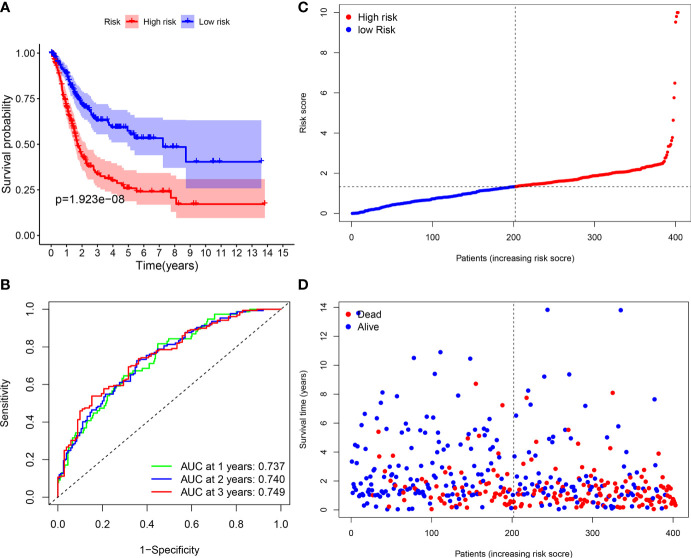
Survival analyses of the signature. **(A)** The Kaplan-Meier survival curve analysis showing the significant difference between the high-risk and the low-risk groups (*p* < 0.05). **(B)** Time-dependent ROC curve analysis showing the significant prognostic value (all AUC value > 0.70). **(C)** Risk score distribution of the patients. **(D)** Survival status scatter plots showing poorer prognosis of the high-risk group compared with the low-risk group.

A total of 406 BLCA patients were then divided into the low-risk group (*n* = 203) and high-risk group (*n* = 203) according to the median risk score (**Figure 8C**). The distribution of survival status of the low-risk group as well as the high-risk group is shown in **Figure 8D**. The area under the curve (AUC) value of ROC for 3-year OS prediction reached 0.749, indicating that the signature had a good prediction for BLCA prognosis (**Figure 8B**). In addition, it was found that the OS of BLCA patients in the high-risk group was obviously worse than that in the low-risk group (**Figure 8A**). The prediction accuracy of the signature in BLCA prognosis was validated in the FAHWMU cohort (*n* = 120). Similarly, BLCA patients at low risk had better OS compared with those at high risk (**Supplementary Figure S2A**). The AUC was 0.793 in the 1st year, 0.752 in the 2nd year, and 0.810 in the 3rd year, respectively (**Supplementary Figure S2B**).

### IGF1 and MMP9 Were Identified as the Hub Genes in the Prognostic Signature

**Figure 9:**
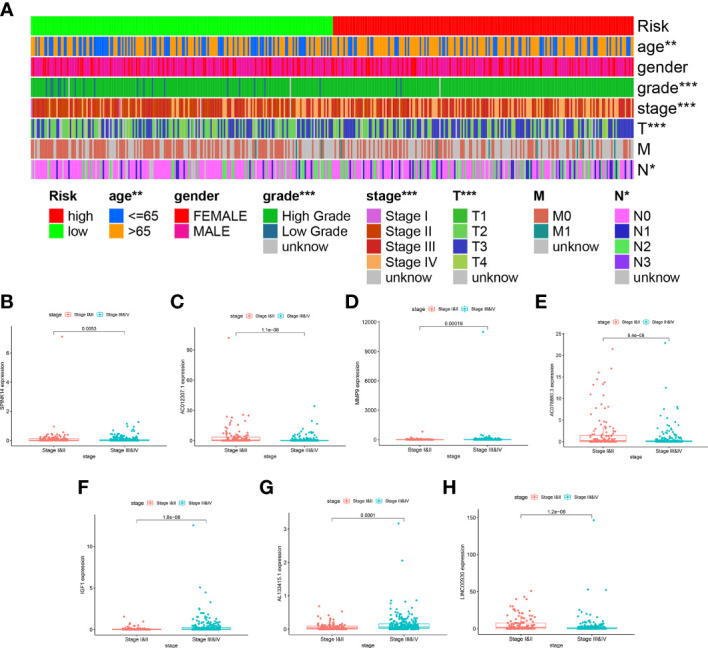
Stage correlation of the risk score and 11 hub genes in the signature. **(A)** Multifactorial heatmap showing certain clinical features with a significant correlation with the risk score. (^*^ *p* < 0.05, ^**^ *p* < 0.01, ^***^ *p* < 0.001) **(B–H)**. The expression of IGF1 and AL133415.1 were positively correlated with TNM stages, while the expression of SPINK14, MMP9, AC078880.3, LINC00930, and AC012307.1 were negatively associated with the TNM stages.

**Figure 10:**
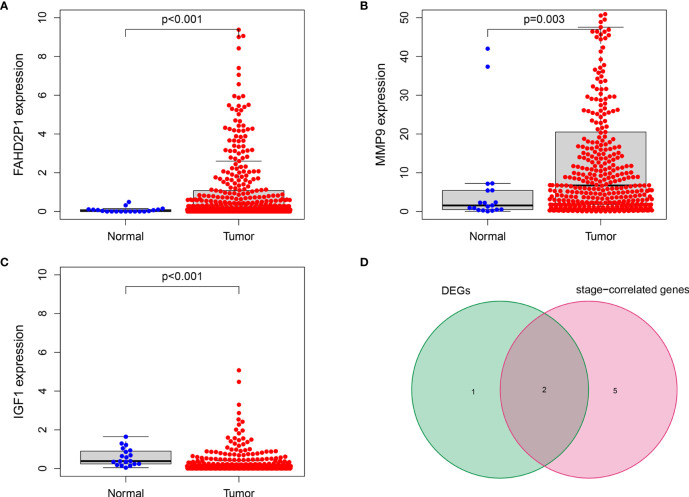
IGF1 and MMP9 were identified as the hub genes in the signature. **(A, B)** The expression of MMP9 and FAHD2P1 was significantly higher in the tumor samples than in the normal samples (*p* < 0.01). **(C)** The expression of IGF1 was significantly lower in the tumor samples compared with the normal samples (*p* < 0.001). **(D)** The Venn plot showing IGF1 and MMP9 as the common genes between DEGs and stage-correlated genes.

As indicated by the clinical clustering heatmap, this TME-related prognostic signature was significantly associated with TNM stage and grade (**Figure 9A**, *p* < 0.001). The correlation analysis showed that IGF1 and AL133415.1 were positively correlated with TNM stage. By contrast, a negative correlation was found between TNM stage and SPINK14 as well as MMP9, AC078880.3, LINC00930, and AC012307.1 (**Figures 9B**–**H**, *p* < 0.01). No significant correlations were found between the TNM stage and other genes, including AC015911.3, ADGRG5, FAHD2P1, and LINC02362 (**Supplementary Figure S3**, *p* > 0.01). Moreover, in comparison with the adjacent nontumorous samples, MMP9 and FAHD2P1 were enhanced in the BLCA samples, whereas IGF1 was reduced (**Figures 10A**–**C**, *p* < 0.01). No significant difference was found in other genes between the BLCA and nontumorous samples (**Supplementary Figure S4**, *p* > 0.01). As shown in the Venn diagram, two common genes (IGF1 and MMP9) were selected through the combination of stage-correlation analyses and differential analyses (*p* < 0.01) (**Figure 10D**). Thus, IGF1 and MMP9 were identified as the hub genes and selected for the next studies.

### IGF1 and MMP9 Were Involved in the Immune Activity of TME in BLCA

**Figure 11:**
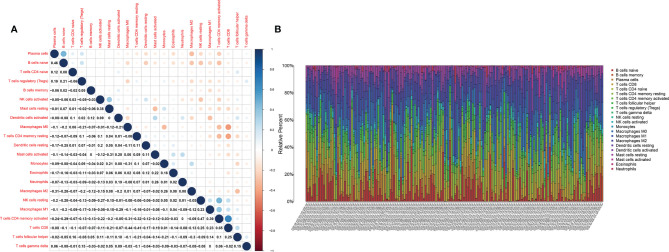
The tumor-infiltrated immune cell profile in BLCA samples and correlation analysis. **(A)** Barplot showing the proportion of 21 types of tumor-infiltrating immune cells in BLCA samples. **(B)** Heatmap showing the correlation between the 22 types of tumor-infiltrating immune cells.

**Figure 12:**
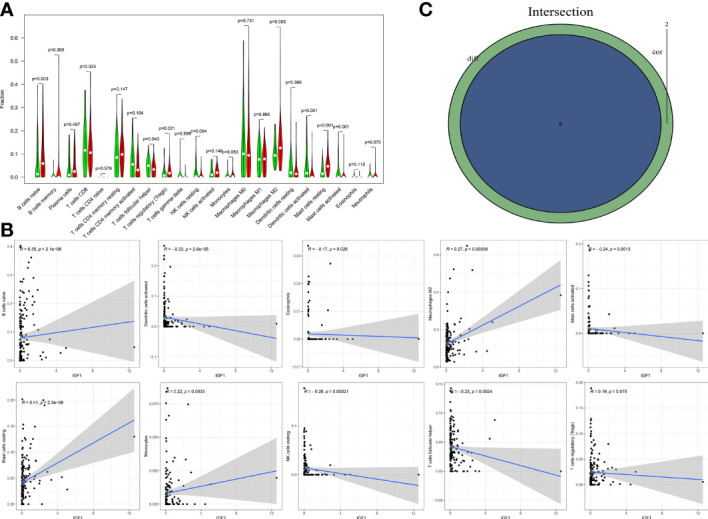
Significant correlation between the expression of IGF1 and the fractions of tumor-infiltrating immune cells. **(A)** Violin plot showing the comparison between 22 types of tumor-infiltrating immune cells with IGF1 expression. **(B)** The correlation tests showing the correlation between 10 types of tumor-infiltrating immune cell proportion with the IGF1 expression (all *p* < 0.05). **(C)** Venn plot showing the collective TICs between the difference and correlation tests.

**Figure 13:**
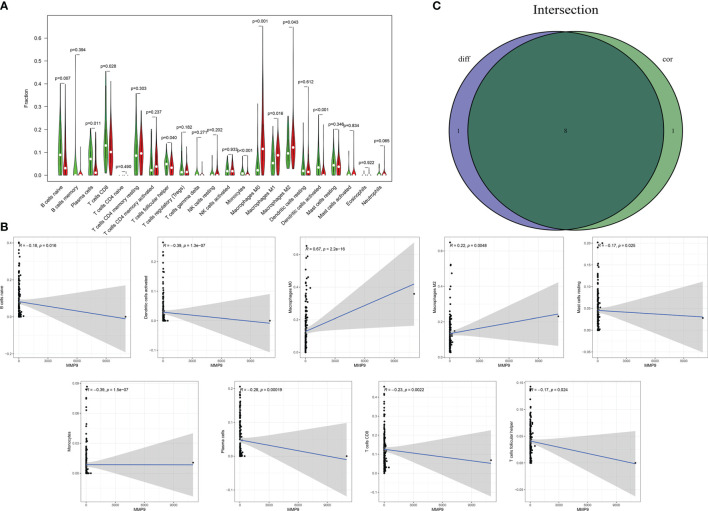
Significant correlation between the expression of MMP9 and the fractions of tumor-infiltrating immune cells. **(A)** Violin plot showing the comparisons between 22 types of tumor-infiltrating immune cells with MMP9 expression. **(B)** The correlation tests showing the correlation between 10 types of tumor-infiltrating immune cells proportion with the MMP9 expression (all *p* < 0.05). **(C)** Venn plot showing the collective TICs between the difference and correlation tests.

The fraction of 22 tumor-infiltrated immune cells in the TCGA cohort was calculated through CIBERSOFT algorithm (**Figures 11A, B**, *p* < 0.05). The intersection of differential analyses revealed that M2 macrophages were positively correlated with the expressions of IGF1 and MMP9. Other immune cells such as B-cell native, were negatively correlated with the expression of MMP9 (*p* = 0.007) and positively associated with the expression of IGF1 (*p* = 0.003) (**Figures 12A**, **13A**). Furthermore, the correlation analyses revealed that several types of immune cells were obviously associated with the expressions of IGF1 and MMP9. With the increase in the expression of IGF1, native B-cell count was increased (*R* = 0.35, *p* < 0.05) (**Figure 12B**). Moreover, MMP9 expression was negatively correlated with the levels of native B cell (*R* = −0.18, *p* < 0.05) (**Figure 13B**). Finally, as shown in the Venn diagram, the common immune cells were selected by the differential analyses and immune cell correlation analyses (**Figures 12C**, **13C**). All these results suggest that IGF1 and MMP9 are closely related to immune infiltrates and may be involved in the immune activity of TME in BLCA.

## Discussion

Recently, the remodeling of the TME has been shown to play a crucial role in the initiation and progression of various human cancers (19). Increasing studies have demonstrated that TME modulation is associated with BLCA progression (20). However, whether the TME status of BLCA contributes to the prediction of BLCA prognosis remains unknown. Recent studies have been reported that the immune and stromal scores in the TME could be calculated by ESTIMATE algorithms, which contributes to provide a novel insight into the development of immune treatment (21). Currently, immunotherapy for TME has been shown to improve BLCA treatment (2). Therefore, more novel candidates are needed for BLCA immunotherapy (22).

In this study, we used the data from the TCGA database to calculate the ESTIMATE, stromal, and immune scores. Our results showed that immune scores and stromal scores were significantly higher in BLCA, associated with lower TNM stages as well as higher stage T levels. The GO and KEGG enrichment analyses indicated that the DEGs were mainly enriched in immune-related pathways. The GSEA analysis confirmed that three hub genes including DCN, IGF1, and MMP9, had a strong correlation with the immune pathway. Moreover, survival analyses preliminarily revealed that the signature could accurately predict the prognosis of BLCA patients. With the aid of the FAHWMU cohort, the clinical value of our prognostic signature was further confirmed.

Recently, TME-related signatures have been identified and validated in multiple human cancers. Huang et al. identified a novel long noncoding RNA signature to improve the prognosis and immunotherapy response of patients with hepatocellular carcinoma (23). Using multiomics analysis, Shi et al. determined a TME-related signature to identify distinct prognostic patterns in osteosarcoma (24). Similarly, the signature correlated with TME was also constructed in the renal cancer to improve immunotherapeutic and antitumor approaches (25). Compared with previous studies, the novel 11 TME-related gene prognostic signature determined in this study has many advantages. The prognostic signature showed a good prognostic value, which was further confirmed in an external BLCA cohort. In addition, the risk scores of the signature were found to be an independent prognostic factor for BLCA treatment. Moreover, as the hub genes in the signature, IGF1 and MMP9 were significantly correlated with immune infiltration. Taken together, our results suggest that this novel TME-related prognostic signature may provide a new theoretical basis for the prognostic assessment of patients with BLCA.

Herein, IGF1 and MMP9 were confirmed as the independent prognostic indicators for BLCA. IGF1, a polypeptide that contributes to tumor progression, has been reported to be involved in TME status (26). Young et al. found that the low expression of IGF1 could induce the aging of hematopoietic stem cells (26). Shi et al. found that IGF1 is associated with the expressions of immune cells, such as NK cells (27). In this study, we found that IGF1 may contribute to an increase in the levels of native B cell and M2 macrophages. MMP9, as a classical immune-related gene, has been demonstrated to play a vital role in the immune treatment of BLCA (28). MMP9 contributes to the promotion of the neutrophil-mediated inflammation, indicating its roles in immune infiltrates (29). Consistent with the previous studies, we found that MMP9 plays a crucial role in the regulation in the activities of immune cells such as native B cells and M0 macrophages, etc. Furthermore, using the STRING database, the potential functional link between TME-related genes and IGF1 as well as MMP9 was obtained (**Supplementary Figure S5**). Combined with these, IGF1 and MMP9, the hub genes of our prognostic signature, may participate in the progression of BLCA *via* the immune activity of TME.

However, there are many limitations in this study. The prognostic value of this signature still needs to be verified in clinical samples with large numbers. In addition, the underlying mechanisms of the 11 TME-related hub genes in immune infiltrates of BLCA should be explored in the future.

In conclusion, we disclose a novel 11 TME-related genes prognostic signature for BLCA, which may provide a new theoretical basis for the prognosis of BLCA patients.

## Data Availability Statement

The original contributions presented in the study are included in the article/**Supplementary Material**. Further inquiries can be directed to the corresponding authors.

## Ethics Statement

The studies involving human participants were reviewed and approved by the Human Research Ethics Committee of The First Affiliated Hospital of Wenzhou Medical University. The patients/participants provided their written informed consent to participate in this study. Written informed consent was obtained from the individual(s) for the publication of any potentially identifiable images or data included in this article.

## Author Contributions

JC and CL designed the study and analyzed the data. JC, BL, YZ, KZ, and RZ revised the images. JC, BC, and YJ performed the literature search and collected data for the manuscript. JZ and YL revised the manuscript. All authors listed have made a substantial, direct, and intellectual contribution to the work and approved it for publication.

## Funding

The project was supported by Wenzhou Medical University Basic Scientific Research (No. KYYW201904).

## Conflict of Interest

The authors declare that the research was conducted in the absence of any commercial or financial relationships that could be construed as a potential conflict of interest.

## Publisher’s Note

All claims expressed in this article are solely those of the authors and do not necessarily represent those of their affiliated organizations, or those of the publisher, the editors and the reviewers. Any product that may be evaluated in this article, or claim that may be made by its manufacturer, is not guaranteed or endorsed by the publisher.

## Supplementary Material

Supplementary Figure 1 The correlations of other clinicopathological characteristics with scores. **(A)** The distribution of score in age. **(B)** The distribution of scores in M classification. **(C)** The distribution of scores in M classification. **(D)** The distribution of score in TNM stage.

Supplementary Figure 2 Survival analysis of the external cohort. **(A)** Kaplan-Meier survival curve of high- and low-risk patients. **(B)** ROC curve of the signature at 1^st^, 2^nd^ and 3^rd^ year.

Supplementary Figure 3 The correlations between the other genes and TNM stage were not significant (*P* > 0.01).

Supplementary Figure 4 The expression levels of the other genes in the signature between BLCA samples and normal samples (*P* > 0.01).

Supplementary Figure 5 The network regarding the potential functional link between TME-related genes and IGF1 as well as MMP9.

## Abbreviations

BLCA, bladder urothelial carcinoma; TME, tumor microenvironment; TCGA, The Cancer Genome Atlas; LASSO, least absolute shrinkage and selection operator; DEGs, differentially expressed genes; OS, overall survival; AUC, area under the curve; ROC, receiver operating characteristic; PPI, protein–protein interaction; GSEA, Gene Set Enrichment Analysis; GO, Gene Ontology; KEGG, Kyoto Encyclopedia of Genes and Genomes.
