# Supplementary figures and images for: Comprehensive Exploration of Tumor Microenvironment Modulation Based on the ESTIMATE Algorithm in Bladder Urothelial Carcinoma Microenvironment
Source: Front Oncol. 2022 Feb 14;12:724261. doi: 10.3389/fonc.2022.724261 (PMC8882770; doi:10.3389/fonc.2022.724261)

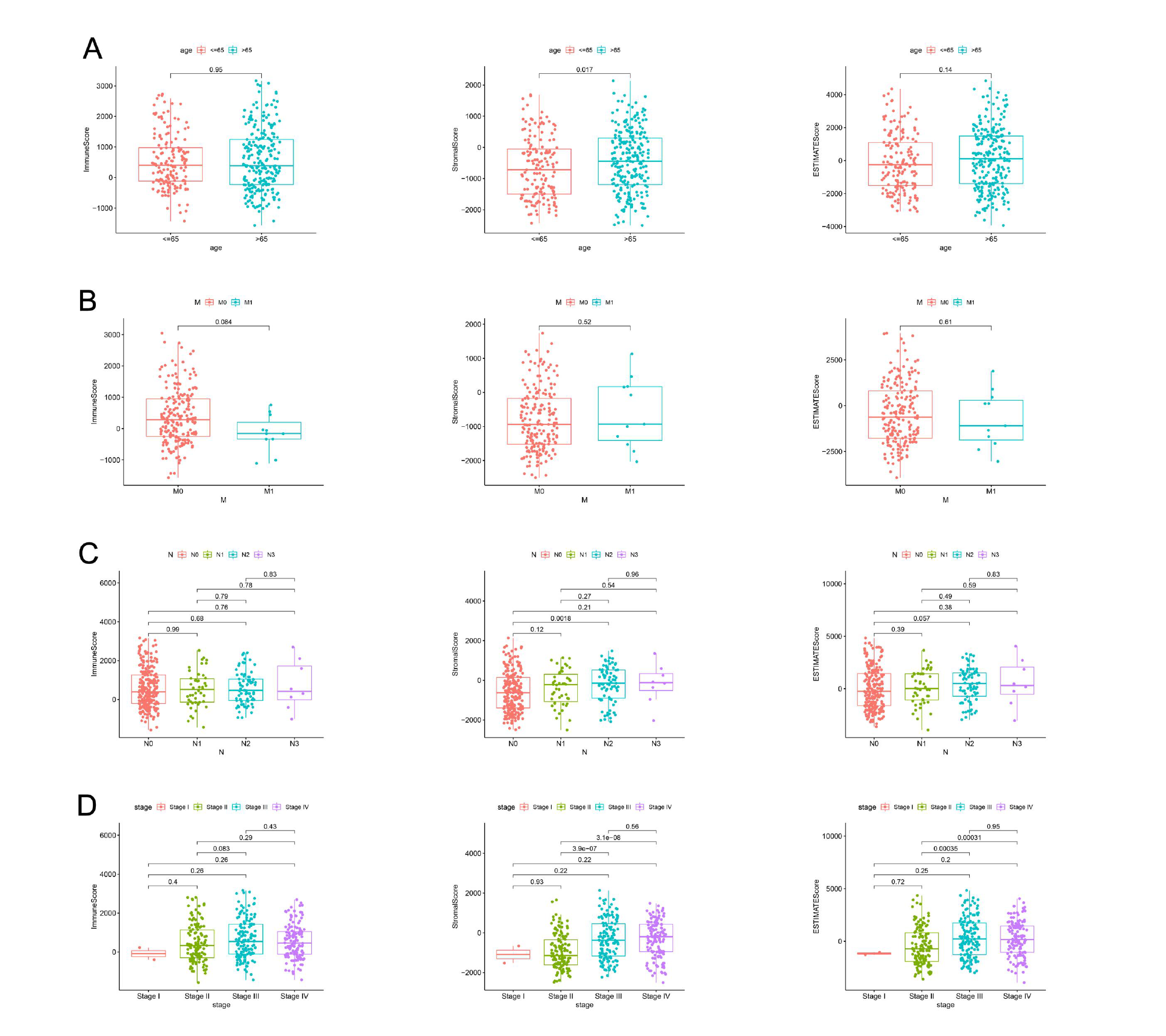

Supplement: Supplementary Figure 1 — The correlations of other clinicopathological characteristics with scores. (A) The distribution of score in age. (B) The distribution of scores in M classification. (C) The distribution of scores in M classification. (D) The distribution of score in TNM stage. [file Image_1.tif]

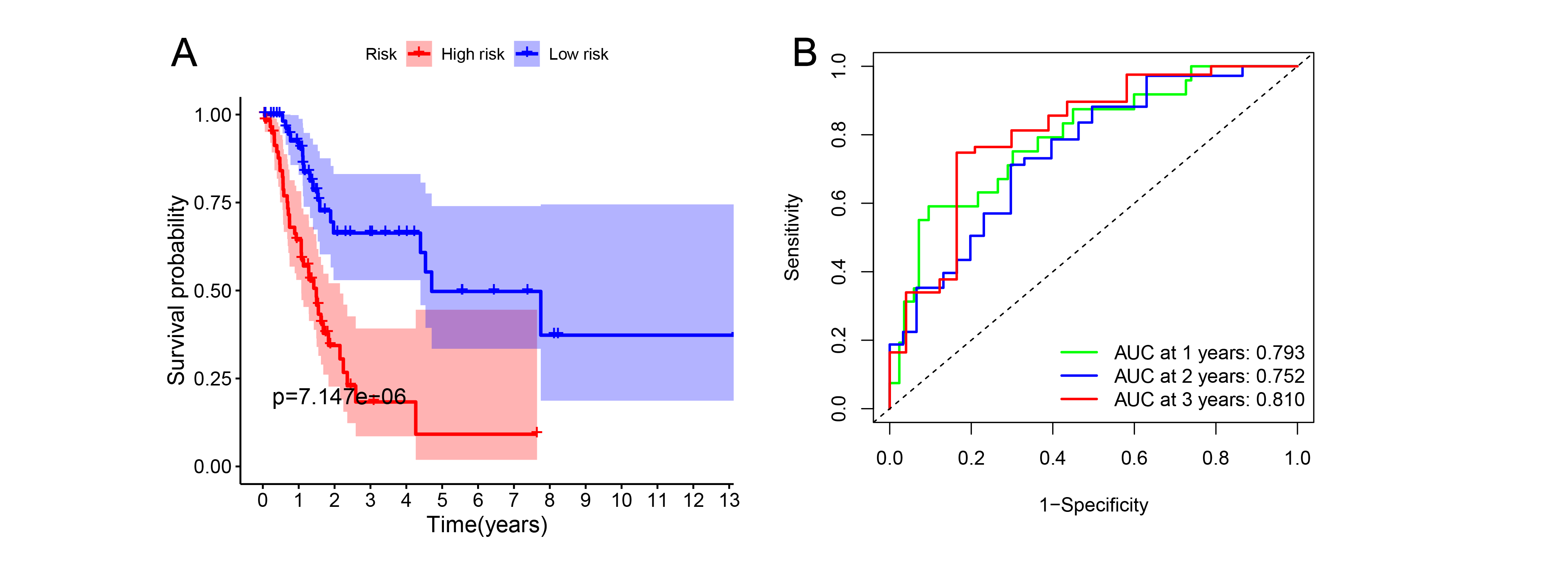

Supplement: Supplementary Figure 2 — Survival analysis of the external cohort. (A) Kaplan-Meier survival curve of high- and low-risk patients. (B) ROC curve of the signature at 1st, 2nd and 3rd year. [file Image_2.tif]

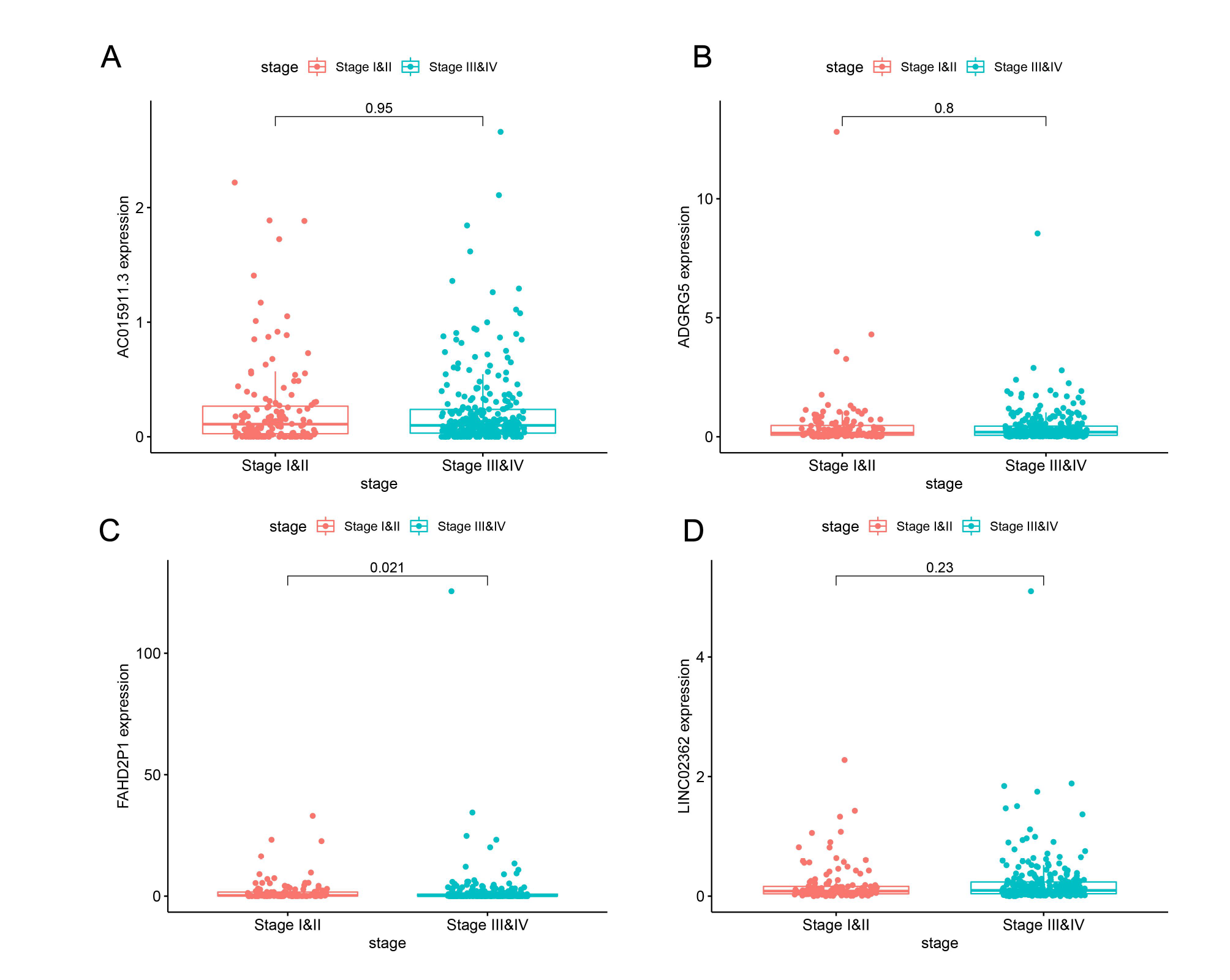

Supplement: Supplementary Figure 3 — The correlations between the other genes and TNM stage were not significant (P > 0.01). [file Image_3.tif]

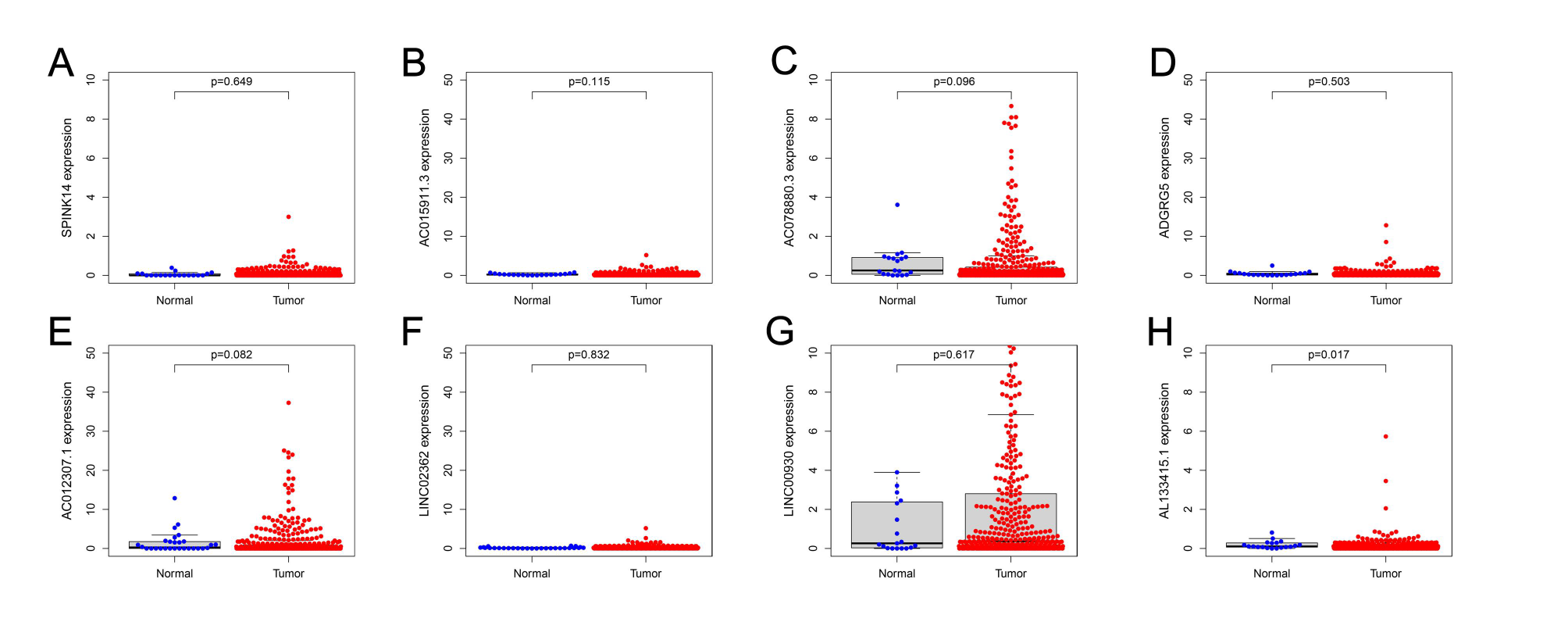

Supplement: Supplementary Figure 4 — The expression levels of the other genes in the signature between BLCA samples and normal samples (P > 0.01). [file Image_4.tif]

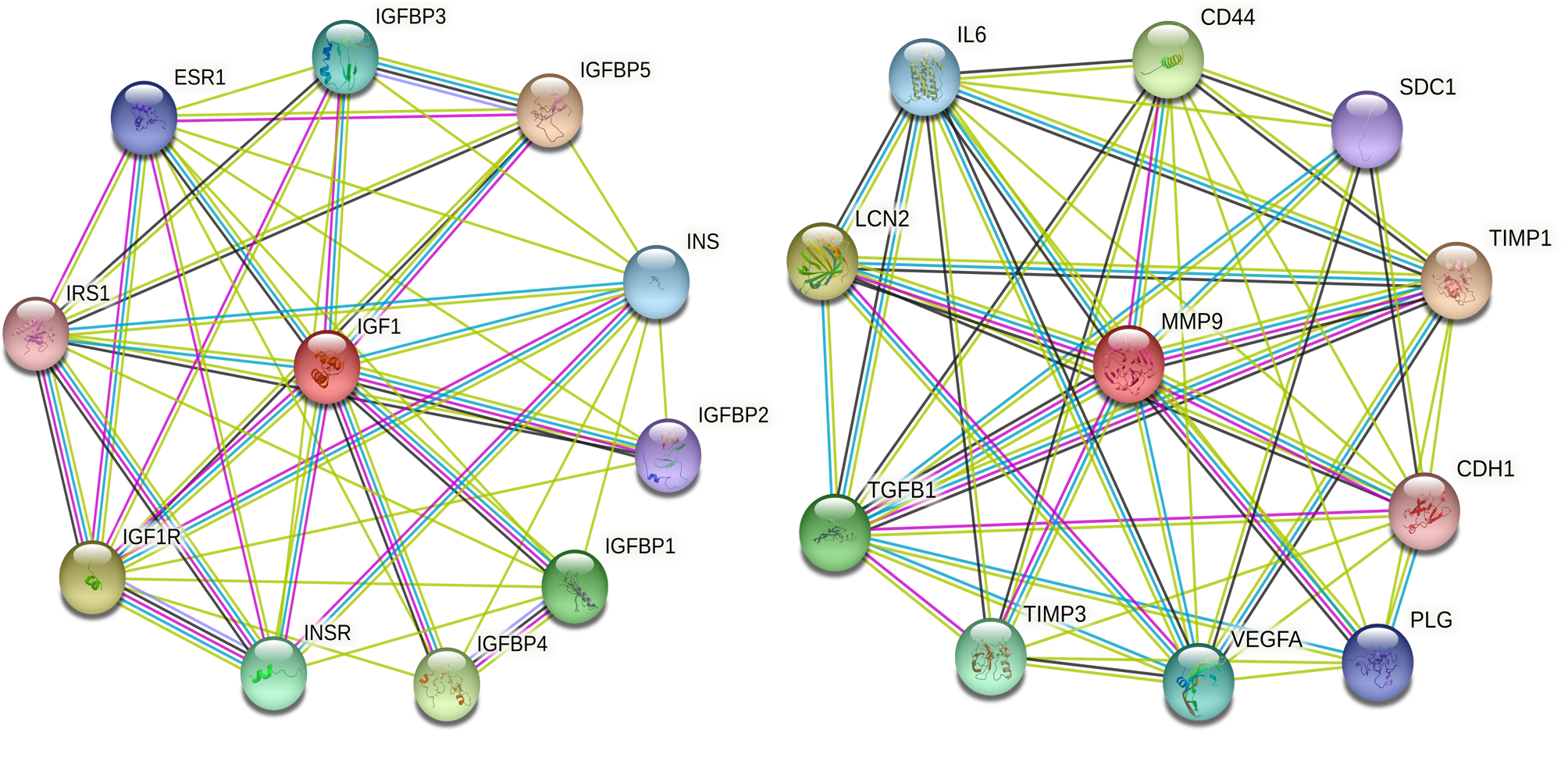

Supplement: Supplementary Figure 5 — The network regarding the potential functional link between TME-related genes and IGF1 as well as MMP9. [file Image_5.tif]
